# Selective Intermittent Preventive Treatment of Vivax Malaria: Reduction of Malaria Incidence in an Open Cohort Study in Brazilian Amazon

**DOI:** 10.1155/2013/310246

**Published:** 2013-03-21

**Authors:** Tony Hiroshi Katsuragawa, Luiz Herman Soares Gil, Alzemar Alves de Lima, Elci Marlei Freitag, Tatiana Marcondes dos Santos, Maria Teixeira do Nascimento Filha, Alcides Procópio Justiniano dos Santos Júnior, Josiane Mendes da Silva, Aline de Freitas Rodrigues, Mauro Shugiro Tada, Cor Jesus Fernandes Fontes, Luiz Hildebrando Pereira da Silva

**Affiliations:** ^1^Instituto de Pesquisas em Patologias Tropicais (IPEPATRO), Rua da Beira, 7671, Bairro Lagoa, 76.812-245 Porto Velho, RO, Brazil; ^2^Fundação Oswaldo Cruz (FIOCRUZ Rondônia), Rua da Beira, 7671, Bairro Lagoa, 76.812-245 Porto Velho, RO, Brazil; ^3^Centro de Pesquisa em Patologias Tropicais (CEPEM/SESAU), Av. Guaporé, 215, Bairro Lagoa, Porto Velho, RO, Brazil; ^4^Universidade Federal de Rondônia (UNIR), Núcleo de Saúde, NUSAU, Campus BR 364, km 9,5, Porto Velho, RO, Brazil; ^5^Universidade Federal de Mato Grosso (UFMT), Núcleo de Estudos de Doenças Infecciosas e Tropicais de Mato Grosso, Rua Luiz Phellipe Pereira Leite s/n, Alvorada, Cuiabá, MT, Brazil

## Abstract

In children, the Intermittent Preventive Treatment (IPTc), currently called Seasonal Malaria Chemoprevention (SMC), was considered effective on malaria control due to the reduction of its incidence in Papua New Guinea and in some areas with seasonal malaria in Africa. However, the IPT has not been indicated because of its association with drug resistance and for hindering natural immunity development. Thus, we evaluated the alternative IPT impact on malaria incidence in three riverside communities on Madeira River, in the municipality of Porto Velho, RO. We denominate this scheme Selective Intermittent Preventive Treatment (SIPT). The SIPT consists in a weekly dose of two 150 mg chloroquine tablets for 12 weeks, for adults, and an equivalent dose for children, after complete supervised treatment for *P. vivax* infection. This scheme is recommend by Brazilian Health Ministry to avoid frequent relapses. The clinic parasitological and epidemiological surveillance showed a significant reduction on vivax malaria incidence. The results showed a reduction on relapses and recurrence of malaria after SIPT implementation. The SIPT can be effective on vivax malaria control in localities with high transmission risk in the Brazilian Amazon.

## 1. Introduction

In Brazil 99% of malaria cases occur in Amazon areas. In recent years, the number of cases per year decreased from 500.000 (2006) to 300.000 (2011) [1]. The Artemisin-based Combination Therapy, used by Brazilian Health Ministry decreased the falciparum malaria incidence (i.e., number of total positive thick blood smear/falciparum thick blood smear × 100) from 26.5 in 2006 to 12.4 in 2011. These results do not consider Asymptomatic *Plasmodium* Carriers (APC), found frequently in holoendemic areas of Africa and Papua New Guinea [2, 3], but not in Amazon areas [4, 5]. However, recent studies show the presence of APC in several areas of the Brazilian Amazon [6–11].

Several works showed that residual malaria may resist to conventional treatment [12–15]. Thus, the APC presence, associated with ineffective tools for vector control and population mobility in Amazon, can be regarded as important factors to explain the increase and maintenance of malaria [16]. Frequencies from 10% to 40% APC were found in adults living in different riverside areas of Madeira River, in Rondônia state [8, 10, 12, 14]. In the same areas, relapses frequency of 6.5% was found after complete treatment with chloroquine (CQ) plus primaquine (PQ) for 14 days, and of 26.7% after the same treatment for 7 days [17]. Previous works were performed to verify the impact of treatment of APC. The treatment of APC of *P. falciparum* led to reduction of malaria cases. However, for APC of *P. vivax* no influence in malaria cases was reported [18]. The ineffectiveness can be attributed to relapses that occur after the complete treatment (CQ plus PQ) of symptomatic patients and APC. Thus, in these areas the clinical immunity observed to *P. vivax* is not species specific but only strain specific [15].

Recently in Papua New Guinea and in African areas where malaria is seasonal, Intermittent Preventive Treatment (IPT) led to a significant reduction on malaria incidence in children [19–24]. World Health Organization currently called Seasonal Malaria Chemoprevention (SMC). Thus, the present work evaluates the introduction of Selective Intermittent Preventive Treatment for malaria control in riverside communities of Madeira River, in Rondônia state, Brazil. In these areas *P. falciparum* corresponds to 10% of malaria cases, while *P. vivax* is the main parasite found in the patients and remains with high prevalence levels [1]. 

Previous studies showed that APC of *P. vivax* prevalence was 20% in these areas [16], and the authors decided to associate Selective Intermittent Preventive Treatment (SIPT). It consisted in a supervised weekly dose of CQ tablets (maximum 300 mg), for 12 weeks. This method was applied selectively to symptomatic and asymptomatic carriers of vivax malaria, after the complete treatment, supervised by a health agent, with CQ plus PQ. This weekly treatment scheme with CQ is recommended by Brazilian Health Ministry in order to avoid relapses in patients after complete treatment, especially in newborns and pregnant women. The dosage has to be proportional to age/weight of the patient.

The main difference between the original IPT [25–30] and the SIPT is the complexity of the composition of the drug required for chemoprophylaxis of falciparum and vivax malaria in high and holoendemic areas of Papua New Guinea and of the sub-Saharan Africa, and also the simplicity of the preventive treatment with CQ, which prevents recurrence. The SIPT was applied after the complete supervised treatment of vivax malaria, and monitoring of clinical cases and APC to prevent recurrences and relapses.

## 2. Materials and Methods

### 2.1. Study Area

**Figure 1:**
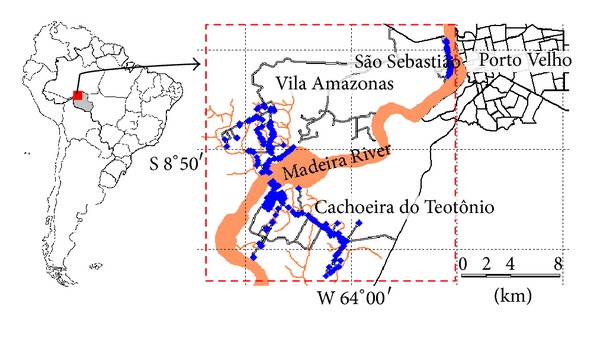
The communities' location in the study area: Vila Amazonas, Cachoeira do Teotônio, and São Sebastião. The blue points indicate the dwellings.

The study was conducted from January 2008 to December 2012 in three riverside communities: Cachoeira to Teotônio (CT), Vila Amazonas (VA), and São Sebastião (SS), located near the municipality of Porto Velho, RO, in Brazilian Amazon (Figure 1) [13, 15, 16]. A Garmin eTrex-Vista was used to georeference the houses. Information from 2007 was obtained for the study baseline.

### 2.2. Study Design

The conventional classification of malaria cases (Brazilian Health Ministry) was adopted in this study. Thus, (i) recrudescence—reappearance of parasitemia by *P. vivax *between 7 to 28 days after the apparent cure of the treated patient, (ii) relapse—reappearance parasitemia by *P. vivax* between 29 to 60 days after the apparent cure, and (iii) reinfection—reappearance of parasitemia after 60 days of apparent cure. In this work, the term recurrence includes relapses and recrudescence. The present work follows an open cohort study, started in January 2008 with a sociodemography census to the total population in the chosen areas. In April 2008 and June 2009, a sectional transversal study was conducted to update the clinical-epidemiological situation of malaria. To determine the prevalence of APC, thick blood smear and PCR exams were performed in all inhabitants, plus by followup performed until june 2009, to all positive PCR and/or negative thick blood smear and malaria symptom absence. The PCR followed the Snounou protocol with modifications [16, 31]. During the study treatment for both falciparum and vivax malaria was systematically performed in all symptomatic patients. It followed the treatment recommended by Brazilian Health Ministry [32]. Falciparum and vivax APC in VA and CT received the treatment in June 2009 and in July 2009, respectively. The Selective Intermittent Preventive Treatment's (SIPT) introduction occurred immediately after APC treatment in VA (June 2009) and was performed until December 2012. In CT, the SIPT was introduced in August 2010 and was performed until December 2012. SS, which was the control community, did not receive SIPT. The conventional treatment was provided only to symptomatic patients.

### 2.3. Malaria Surveillance

The malaria surveillance was done in the communities by our trained health agents, on a weekly basis, throughout the study period. The inhabitants were surveyed about fever episodes or any malaria symptoms. When it was suspected that a villager had malaria, a search for *Plasmodium* was performed, either by thick blood smear or/and PCR [11, 15]. The thick blood smear samples were analyzed by technicians of the Centro de Pesquisa em Medicina Tropical (CEPEM) for positive or negative. The asymptomatic or symptomatic carriers of *P. vivax* were monitored for eventual relapses. Malaria incidence was calculated in percentage (number of cases per 100 people). The cases presented at health facilities were supervised by our health agents. The asymptomatic relapses were not considered.

### 2.4. Complete Treatment

The symptomatic inhabitants were treated according to the Brazilian Health Ministry protocol [26]. Thus, *P. falciparum* infection was treated with artemether-lumefantrine, Coartem [32, 33], while *P. vivax* infection was treated with CQ (25 mg/kg base over 3 days: 10.0 mg/kg on day 1, and 7.5 mg/kg on days 2 and 3) plus PQ (0.5 mg/kg/day for 7 days) for seven days (short scheme), and adjusted for patient weight [32]. The treatments started between 24 and 48 hours after the first symptom episode or positive laboratory diagnosis. PQ was not administered in pregnant women and newborns younger than six-months-old, and no allergic cases were related throughout the study.

### 2.5. Selective Intermittent Preventive Treatment

The SIPT is based on weekly CQ dose (5.0 mg/kg base) for 12 weeks, beginning 7 days after the end of the supervised complete treatment. This scheme is recommended by Brazilian Health Ministry to avoid frequent relapses. The SIPT was applied to APC in VA (June 2009) and CT (August 2010), and all symptomatic cases after these dates with thick blood smear diagnostic.

### 2.6. Entomological Surveillance

Mosquitoes were collected by Human Landing Catches (HLC) from January 2008 to December 2012. The catches were carried out monthly in CT and VA. In a house assigned for HLC, four health workers, two indoors and two outdoors, collected the mosquitoes landing on (safely) exposed legs and feet, from 06:00 pm to 12:00 am. The Brazilian official protocol “*Estratégias de Proteção para o Profissional Técnico Responsável pelas Capturas de Anofelinos*” [32] was followed. The Human Biting Rate (HBR) was calculated [32, 34]. No accidental infection occurred to any health worker during the experiments. The Ethical Committee approval for this study was granted by the Ethical Committee of CEPEM (CEP-CEPEM). The term of agreement for the catching procedures was provided to all health workers involved, who consented to it.

### 2.7. Statistical Analysis

Statistical analyses were performed using GraphPad Prism (version 5.0). For malaria incidence analysis, before and after SIPT application, the Wilcoxon test was used with confidence intervals of 95%. Odds ratio and relative risk were calculated by contingency table for malaria incidence between localities. 

### 2.8. Ethical Considerations

The present study was approved by the Ethical Committee of the CEPEM (CEP-CEPEM), and it was registered under the number 070/2008. Information about the study was provided to the all inhabitants, and a term of agreement was signed.

## 3. Results

### 3.1. Baseline Cross-Section Survey and APC Prevalence

In 2007 a survey was conducted in the localities to get the baseline. A total of 869 inhabitants were counted in the three communities. No difference was found in the both age and gender of inhabitants (*P* = 0.737 and *P* = 0.344, resp.), malaria incidence (*P* = 0.824) or prevalence (*P* = 0.626) of *P. vivax* (Table 1). When considering the inhabitants' living time in endemic areas and the APC prevalence, statistical difference was found amongst the community. The distribution of *P. vivax* and *P. falciparum*, in APC, by gender and age, showed a percent difference (Table 2).

### 3.2. Population Mobility and Migrations

**Figure 2:**
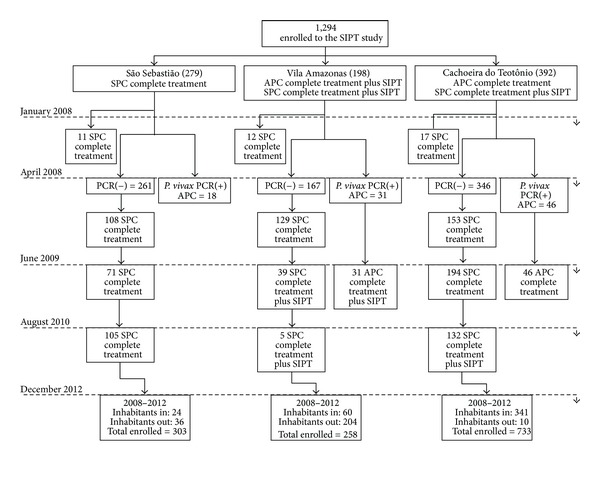
Participant flow. SPC: Symptomatic *Plasmodium *Carriers by *P. vivax*; APC: Asymptomatic *Plasmodium *Carriers by *P. vivax*; SIPT: Selective Intermittent Preventive Treatment.

After 55 months of the open cohort study, of 1,286 informed and consenting subjects, 869 were tested by PCR in 2008 to identify APC of *P. vivax*; 75 and 126 SIPT were performed in VA and CT, respectively (Figure 2). The communities are located in the Madeira River riverbanks, which is affected by the implementation of a hydroelectric power plant. Thus, this area had a higher level of inhabitants' flow during the study, registered by the monthly census. Additionally, survey of malaria incidence allowed estimating cases/100 persons per year, in a six-month period (Table 3). This data contains the relative risk (RR) calculated in SS, considering the population flow in each community. 

### 3.3. Followup of the SIPT and Malaria Incidence

**Figure 3:**
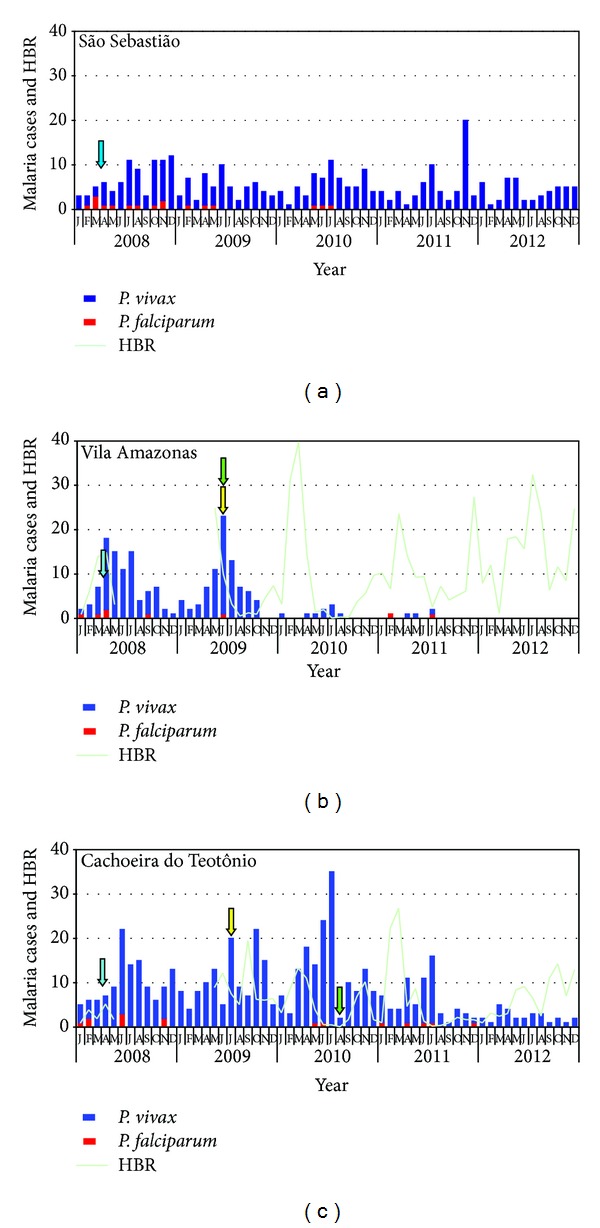
Malaria number cases by *Plasmodium* species in Vila Amazonas, Cachoeira do Teotônio, and São Sebastião from January/2008 to December/2012. The mosquitoes' collection was evaluated by Human biting rate (HBR) only in Vila Amazonas and Cachoeira do Teotônio. The blue arrows indicate cross-section survey to identify APC. The yellow arrows indicate the APC treatment. The green arrows indicate SIPT procedure beginning.

**Figure 4:**
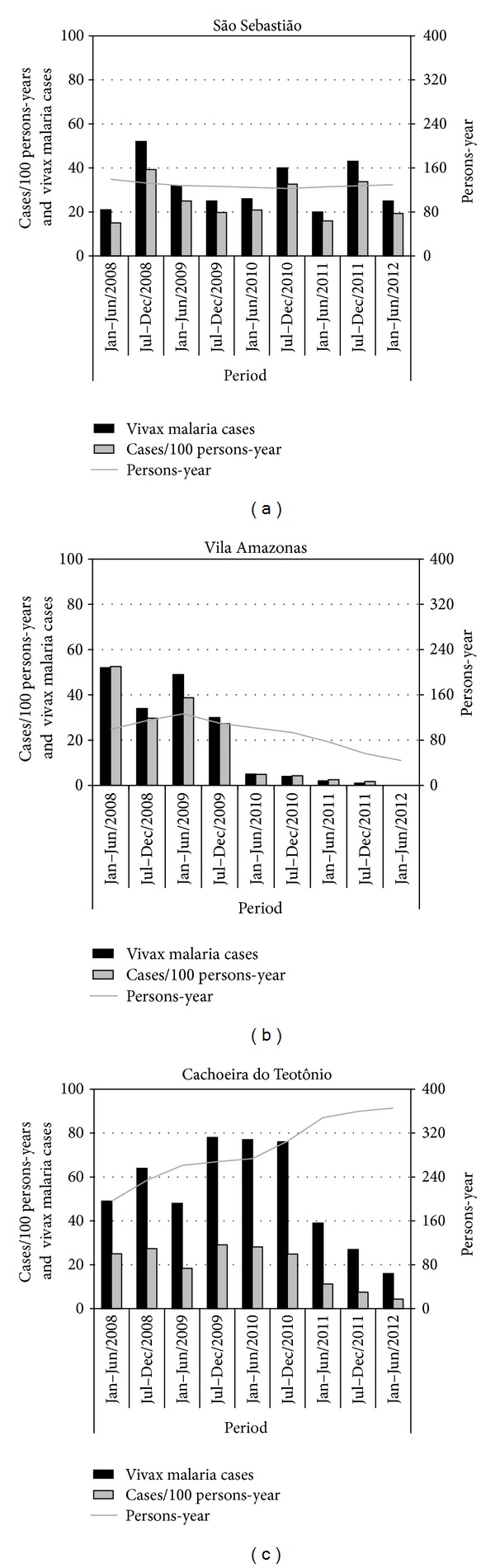
Semiannual distribution of cases/100 persons-year, total vivax malaria cases, and persons-year in the communities from January 2008 to December 2012.

From January 2008 to December 2012 all cases of both symptomatic vivax and falciparum malaria were treated in the communities. Besides, from June to July 2009 all the identified APC in VA and CT received the same treatment. The SIPT introduction for vivax malaria patients started in June 2009, in VA, and August 2010, in CT (Figure 3). In SS the APC did not receive either treatment or SIPT (community of control). After the treatment of APC and SIPT introduction, VA showed a significant reduction in malaria incidence (Table 3). The number of cases/100 persons-year was reduced from 38.7 to 1.8 in five semesters. This reduction was significantly higher than found in SS (*P* < 0.05). CT showed no reduction in vivax malaria incidence two semesters after the treatment of APC. However, after SIPT application for symptomatic vivax malaria, a significant decrease of malaria incidence was observed (*P* < 0.05). The recurrence of malaria in inhabitants during the study suggests that SIPT inhibits relapses. In SS the recurrence percentage has not changed significantly (*P* > 0.05). However, in VA and CT there was a significant decrease after SIPT application (Table 4). Analyzing equal periods before and after the introduction of SIPT (Table 5), a significant reduction was observed in VA (*P* = 0.0144) and CT (*P* = 0.0010) compared to the community of control, SS (*P* = 0.1824; *P* = 0.1248). After the treatment of APC of *P. falciparum* in VA and CT, no clinical cases for 19 and 9 months, respectively, were reported. The results are similar to a previous study [15]. However, this result was also observed in the community of control. The authors consider that the treatment adopted by the Brazilian Health Ministry, with the combination of artemether/lumefantrine as first-line treatment of symptomatic falciparum malaria, is the main responsible for this reduction (Table 4). Both vivax malaria cases and cases/100 persons-year significantly decreased in VA and CT, while in the SS, they presented stable (Figure 4). In the present study, no adverse events related to chloroquine and/or primaquine were reported. The adherence of the participants to SIPT was 100.0% in the VA and 98.5% in the CT.

### 3.4. Seasonal Mosquitoes' Densities

The Human Biting Rate (HBR) was calculated during the study period, with a break, suggested by the Brazilian Health Ministry, between April/2008 and June/2009. In both localities, VA and CT elevations were observed after the rainy season and the application of the SIPT (Figure 3). In the present study more than 99% of the mosquitoes collected were *Anopheles darlingi*.

## 4. Discussion

The success in reducing the incidence of falciparum malaria with Artemisinin-based Combination Therapy (ACT), recommended by the Brazilian Health Ministry [32], did not affect the high incidence of vivax malaria in the Brazilian Amazon. The results of this study demonstrate that the proposed SIPT, to prevent recurrences, provides an efficient solution to control malaria by *P. vivax* in these areas. It is very important to observe the profile of vivax malaria in VA between 2010 and 2012, contrasting with the maximum values of HBR of 39.8 (Mar/2010), 23.6 (Mar/2011), 27.4 (Dec/2011), 32.4 (Jul/2012), and 24.8 (Dec/2012), and the rare clinical episodes of vivax malaria. This is an evidence of the effectiveness of protection provided by the SIPT, which rapidly reduces the sources of infection, even with abundant population of mosquitoes. 

In this study, the very low incidence of falciparum malaria in the Brazilian Amazon allows the use of CQ as preventive treatment to the relapses of malaria by *P. vivax*. A second important difference is that IPT should be applied to all children for a long period, while the SIPT is selectively applied for only 12 weeks, and it is administered immediately after the supervised complete treatment for children and adult patients who had clinical infection by *P. vivax*. The SIPT can be considered an alternative method for the generalized use of CQ and in this case decreases the selective pressure on *Plasmodium*, avoiding the selection of resistant strains. Throughout this study, the decrease in cases of vivax malaria that was observed in VA corresponded to a decrease in the number of CQ tablets distributed in that locality. The number gradually decreased from 860 in 2008, to 790 in 2009, 90 in 2010, 30 in 2011, and 0 in 2012. We observed that the SIPT application immediately after complete treatment for both APC and SPC, reduce more quickly the incidence of vivax malaria.

Our next goal is to verify if SIPT affects the natural development of immunity to vivax malaria. This problem has been studied in procedures using IPT in Africa and Papua New Guinea, with conflicting results. While one study evaluated the incidence of malaria on children in the following year that were treated (IPTc) [19, 35], another suggests that the children may gain some weight [36]. In another study, children, who were treated monthly/bimonthly with IPTc, had interference in the development of protective immunity against clinical malaria by *P. falciparum* [20–24, 27, 37].

A crucial point to the success of the SIPT procedure is the accuracy and efficiency of clinical and epidemiological surveillance. That depends on dedication and competence of health workers, as well as cooperation of local residents, who must have confidence in health workers. This study shows that public education and clarity of the healthcare procedures improves the participation of residents in the intervention [38]. In this study, the health workers got an acceptance rate of 98.3% by the inhabitants. Importantly, the involvement of health workers in this activity consumes less time than what is necessary for fetal-maternal care or mental health. Notably the SIPT can rapidly improve the wellbeing of localities and cause a favorable impact on other health problems. We suggest that it would be useful to organize a mobile team, which would cover a huge area to establish the widespread application of the SIPT. In the present study, the significant reduction in the incidence of vivax malaria with the new procedure reduces the number of individuals who need antimalarial drugs, while minimizing the criticism of IPT procedures. The procedure may be applicable to other tropical areas in Amazon Basin, where recurrence cases and treatment failure in malaria vivax was reported [39, 40]. Also include the identification and treatment of asymptomatic Plasmodium carriers when the approach is malaria control [41, 42].

One last point is the cost effectiveness of the SIPT. There is no doubt about the positive effect observed in this study, which could eradicate malaria in endemic areas of the Brazilian Amazon. With this protocol, the cost effectiveness depends on two elements: (i) improvements and maintenance of basic healthcare, and clinic-epidemiological surveillance, (ii) adequate physical facilities, equipment, and qualified personnel for the diagnosis of malaria, including molecular tools for detection of APC. To be effective, investments shall be made in training and qualifying technical and health workers. Thus, both elements are equally effective in improving healthcare in the Brazilian Amazon, particularly in reducing infant and maternal mortality, and other local endemic diseases which are responsible for high infant and maternal morbidity.

## Figures and Tables

**Table 1:** Demographic characteristics and malaria baseline survey in the study's communities, in 2007 and 2008.

Characteristics	Group	Locality			*P*
		São Sebastião	Vila Amazonas	Cachoeira do Teotônio	
	0–5	32 (11.4)	20 (10.1)	36 (9.2)	
Age group *n* (%)	6–15	73 (26.2)	46 (23.2)	93 (23.7)	0.737
	>15	174 (62.4)	132 (66.7)	263 (67.1)	
	Total	**279**	**198**	**392**	
Gender *n* (%)	Male	149 (53.4)	119 (60.1)	218 (55.6)	0.344
	Female	130 (46.6)	79 (39.9)	174 (44.4)	
Previous malaria cases within age group *n* (%)	None	36 (12.9)	23 (11.6)	61 (15.6)	0.594
	1–4	94 (33.7)	59 (29.8)	121 (30.9)	
	5–10	89 (31.9)	62 (31.3)	113 (28.8)	
	>10	60 (21.5)	54 (27.3)	97 (24.7)	
Living in endemic area, in years, Mean ± SD (CI 95% mean)		22.89 ± 17.92 (20.79–25.00)	13.86 ± 14.23 (11.86–15.85)	7.88 ± 10.81 (6.81–8.95)	<0.001
Malaria incidence in 2007, before the study cases/100 inhabitants (CI 95%)		24.9 (19.7–30.7)	28.5 (22.1–35.6)	26.6 (22.4–31.3)	0.824
*P. vivax* prevalence % (CI 95%)		19.0 (14.4–24.4)	23.8 (17.9–30.6)	21.6 (17.7–26.0)	0.626
*P. vivax* prevalence proportion % (CI 95%)		76.3 (64.2–85.8)	83.7 (71.3–92.1)	81.2 (72.7–87.9)	0.945
*P. vivax* in APC prevalence % (CI 95%)		6.5 (4.0–9.8)	15.7 (11.1–21.2)	11.7 (8.8–15.2)	0.014
*P. falciparum* prevalence % (CI 95%)		5.9 (3.4–9.5)	4.7 (2.2–8.6)	5.0 (3.1–7.6)	0.335
*P. falciparum* prevalence proportion % (CI 95%)		23.7 (14.2–35.8)	16.3 (7.9–28.7)	18.8 (12.1–27.3)	0.715
*P. falciparum* APC prevalence % (CI 95%)		1.1 (0.3–2.9)	6.1 (3.3–10.1)	1.3 (0.5–2.8)	<0.001

SD: standard deviation. CI 95%: confidence interval. *P*: *P* value. APC: asymptomatic *Plasmodium* carrier (Apr/2008).

**Table 2:** Asymptomatic *Plasmodium* carriers' distribution by community, sex, and age group, in April/2008.

			APC age (year)							
Community	Sex	*n*	<15			≥15			Total	%
			*Pv *	*Pf *	PFV	*Pv *	*Pf *	PFV		
	Male	149	2	—	—	9	3	—	14	5.0
São Sebastião	Female	130	3	—	—	4	—	—	7	2.5
	Total	**279**	**5**	—	—	**13**	**3**	—	**21**	**7.5**
	Male	119	—	—	—	22	7	1	30	15.2
Vila Amazonas	Female	79	—	—	—	7	3	1	11	5.6
	Total	**198**	—	—	—	**29**	**10**	**2**	**41**	**20.7**
	Male	218	4	—	—	24	3	—	31	7.9
Cachoeira do Teotônio	Female	174	4	—	—	14	2	—	20	5.1
	Total	**392**	**8**	—	—	**38**	**5**	—	**51**	**13.0**

APC: asymptomatic *Plasmodium* carriers. *n*: population. %: percentage. (—): numeric data equal zero, not rounding result. *Pv*: *Plasmodium vivax*. *Pf*: *Plasmodium falciparum*. PFV: mixed-species malaria.

**Table 3:** *Plasmodium vivax* incidence throughout the follow-up period.

Community	Cohort period	Persons-year	*P. vivax* incidence		RR* (CI 95%)	*P*
			*n*	Cases/100 persons-year		
São Sebastião	Jan–Jun/2008	139.5	21	15.1	—	—
	Jul–Dec/2008	132.5	52	39.2	—	—
	Jan–Jun/2009	128.0	32	25.0	—	—
	Jul–Dec/2009	126.5	25	19.8	—	—
	Jan–Jun/2010	124.5	26	20.9	—	—
	Jul–Dec/2010	122.5	40	32.7	—	—
	Jan–Jun/2011	125.5	20	15.9	—	—
	Jul–Dec/2011	127.5	43	33.7	—	—
	Jan–Jun/2012	129.5	25	19.3	—	—
	Jul–Dec/2012	130.5	24	18.4	—	—

Vila Amazonas	Jan–Jun/2008	99.0	52	52.5	3.489 (2.121–5.901)	<0.0001
	Jul–Dec/2008	114.5	34	29.7	0.757 (0.487–1.164)	0.2064
	Jan–Jun/2009^A^	126.5	49	38.7	1.549 (0.994–2.440)	0.0530
	Jul–Dec/2009	110.0	30	27.3	1.380 (0.810–2.367)	0.2367
	Jan–Jun/2010	101.5	5	4.9	0.236 (0.081–0.582)	0.0009
	Jul–Dec/2010	93.5	4	4.3	0.131 (0.040–0.338)	<0.0001
	Jan–Jun/2011	77.0	2	2.6	0.163 (0.026–0.600)	0.0031
	Jul–Dec/2011	56.5	1	1.8	0.052 (0.003–0.270)	<0.0001
	Jan–Jun/2012	44.0	0	—	0.000 (0.000–0.391)	0.0009
	Jul–Dec/2012	42.5	0	—	0.000 (0.000–0.408)	0.0052

Cachoeira do Teotônio	Jan–Jun/2008	196.0	49	25.0	1.661 (1.004–2.820)	0.0479
	Jul–Dec/2008	234.0	64	27.4	1.217 (0.844–1.761)	0.2941
	Jan–Jun/2009	261.0	48	18.4	0.736 (0.471–1.160)	0.1828
	Jul–Dec/2009^B^	268.0	78	29.1	1.473 (0.948–2.349)	0.0867
	Jan–Jun/2010	273.5	77	28.2	1.348 (0.872–2.136)	0.1850
	Jul–Dec/2010^C^	305.5	76	24.9	0.762 (0.521–1.126)	0.1683
	Jan–Jun/2011	348.0	39	11.2	0.703 (0.413–1.227)	0.2070
	Jul–Dec/2011	359.5	27	7.5	0.223 (0.136–0.359)	<0.0001
	Jan–Jun/2012	365.5	16	4.4	0.236 (0.123–0.444)	<0.0001
	Jul–Dec/2012	374.0	12	3.2	0.175 (0.084–0.346)	<0.0001

*n*: Incident cases. RR: relative risk. *P*: *P* value. *incidence density ratio, considering São Sebastião data as a control community. ^A^APC treatment and SIPT beginning (June/2009). ^B^APC treatment (July/2009).^ C^SIPT beginning (August/2010).

**Table 4:** Recurrence frequency in the same dwellers throughout the follow-up period.

				Clinical vivax malaria recurrence in the same dweller							
Community	Period/year	*Pf *	*Pv *	APC			SPC			Total	%
				1	2	3	1	2	3		
São Sebastião	Jan–Jun/2008	6	21	—	—	—	12	3	1	5	23.8
	Jul–Dec/2008	5	52	—	—	—	29	7	3	13	25.0
	Jan–Jun/2009	3	32	2	1	—	16	3	2	8	25.0
	Jul–Dec/2009	—	25	4	—	—	10	4	1	6	24.0
	Jan–Jun/2010	2	26	3	2	1	8	1	2	9	34.6
	Jul–Dec/2010	1	40	3	—	—	20	7	1	9	22.5
	Jan–Jun/2011	—	20	3	—	—	9	4	—	4	20.0
	Jul–Dec/2011	—	43	2	2	1	19	6	1	12	27.9
	Jan–Jun/2012	—	25	3	1	—	9	4	1	7	28.0
	Jul–Dec/2012	—	24	1	—	1	10	5	—	7	29.2

Vila Amazonas	Jan–Jun/2008	4	52	—	—	—	25	12	1	14	26.9
	Jul–Dec/2008	1	34	—	—	—	23	4	1	6	17.6
	Jan–Jun/2009^A^	1	49	2	—	—	34	5	1	7	14.3
	Jul–Dec/2009	—	30	1	—	—	23	3	—	3	10.0
	Jan–Jun/2010	—	5	—	—	—	5	—	—	—	—
	Jul–Dec/2010	—	4	—	—	—	4	—	—	—	—
	Jan–Jun/2011	1	2	—	—	—	2	—	—	—	—
	Jul–Dec/2011	1	1	—	—	—	1	—	—	—	—
	Jan–Jun/2012	—	—	—	—	—	—	—	—	—	—
	Jul–Dec/2012	—	—	—	—	—	—	—	—	—	—

Cachoeira do Teotônio	Jan–Jun/2008	6	49	1	—	—	34	7	—	7	14.3
	Jul–Dec/2008	2	64	4	—	—	39	6	3	12	18.8
	Jan–Jun/2009	—	48	3	1	—	33	5	—	6	12.5
	Jul–Dec/2009^B^	—	78	6	—	—	39	9	5	19	24.4
	Jan–Jun/2010	2	77	3	—	—	47	12	1	14	18.2
	Jul–Dec/2010^C^	—	76	2	1	—	39	15	1	18	23.7
	Jan–Jun/2011	3	39	—	—	—	27	6	—	6	15.4
	Jul–Dec/2011	2	27	2	—	—	21	2	—	2	7.4
	Jan–Jun/2012	—	16	1	—	—	15	—	—	—	—
	Jul–Dec/2012	—	12	1	—	—	11	—	—	—	—

*Pf*: *Plasmodium falciparum* incidence; *Pv*: *Plasmodium vivax* incidence. APC: asymptomatic *Plasmodium* carrier. SPC: symptomatic *Plasmodium* carrier. 1, 2 or 3: number of clinical vivax malaria episode in the same dweller (the first episode “1” is a primary episode). Total: total number of recurrence, excluding the primary episode. %: percent relative to incident cases. ^A^APC treatment and SIPT beginning (June/2009). ^B^APC treatment (July/2009). ^C^SIPT beginning (August/2010). (—): numeric data equal zero, not rounding result.

**Table 5:** Statistical analysis of malaria incidence in the communities, before and after SIPT application.

Community		Period	*P. vivax* incidence			
			*n*	Mean	SE	*P*
Vila Amazonas	Before	Jun/07 to May/09	145	6.042	1.015	0.0144
	After	Jun/09 to May/11	65	2.708	1.079	

Cachoeira do Teotônio	Before	Aug/08 to Jul/10	292	12.170	1.528	0.0010
	After	Aug/10 to Jul/12	130	5.417	0.849	

São Sebastião	Before	Jun/07 to May/09	145	6.042	0.663	0.1824
	After	Jun/09 to May/11	118	4.917	0.542	
	Before	Ago/08 to Jul/10	145	6.042	0.674	0.1248
	After	Aug/10 to Jul/12	120	5.000	0.819	

Before: before SIPT application. After: after SIPT application. SE (standard error) and mean: Mann-Whitney test. *P*: *P* value. Wilcoxon rank test for pared data analysis. São Sebastião is a control community. No SIPT was applied.
